# Intergenerational Learning Processes of Traditional Medicinal Knowledge and Socio-Spatial Transformation Dynamics

**DOI:** 10.3389/fsoc.2022.661992

**Published:** 2022-07-07

**Authors:** Anne Ouma

**Affiliations:** Department of Epidemiology and Global Health, Vaartoe Centre for Sami Research, Umeå University, Umeå, Sweden

**Keywords:** migration, youth, socio-spatial transformation, urbanization, inter generational learning, traditional medicinal knowledge

## Abstract

The transfer of traditional knowledge to new generations of traditional medicinal practitioners takes place through place-based intergenerational learning processes, which are increasingly challenged by intensified rural–urban migrations and accelerating biodiversity loss. Research on traditional medicinal knowledge (TMK) has mainly focused on the medicinal properties of different plant species while social, economic, and locational aspects of TMK learning processes have received less attention. The purpose of this article is to contribute to the research field by examining how the learning processes of TMK are affected by on-going socio-spatial transformations in rural and urban parts of the Eastern Lake Victoria region. Urbanization and migration are transforming the learning processes of TMK and affect the ways traditional practitioners are able to transfer TMK to a new generation of practitioners. Based on in-depth interviews, participant observations and focus group discussions with male and female traditional practitioners aged between 30 and 95 from rural and urban settings in Mwanza (Tanzania) and Nyanza (Kenya) in the Eastern Lake Victoria Region. The study analyzes the role of socio-spatial and migration dynamics on major intergenerational forms of learning of TMK (learning in place; being sent; ritual places); health knowledge diffusion and interactions between TMK and formal health systems. Despite some major challenges to the continuity of TMK learning due to increased migration identified by the traditional practitioners, many also saw emerging roles for TMK in primary health care for sustainable livelihoods for the younger generations of men and women in this region.

## Introduction

Lake Victoria region has been characterized by rapid socio-economic and environmental changes during the last few decades. Many people in western Tanzania and western Kenya are dependent on the Lake Victoria ecosystem for their livelihoods and have to struggle with the consequences of environmental degradation, loss of biological diversity, and unemployment. A notable trend is an increase in rural–urban migration of the youth in recent decades (De Bruijn et al., 2001; Helgesson, 2006) as well as a rapidly emerging urban market economy for traditional medicinal products in this region.

Similar to other parts of the world, within this study area, inter-generational learning is understood within a context of a system of pedagogy in existence for millennia and viewed by some to be as old as mankind (Hoff, 2007). The World Health Organization defines traditional knowledge transmission as often relying exclusively on practical experience and observations handed down from generation to generation [World Health Organization (WHO), 2002]. Traditional medicinal knowledge is widely discussed as a knowledge system on health and wellbeing with temporal and spatial dimensions with its own epistemological, philosophical, scientific, and logical validity (Battiste and Henderson, 2000). Traditional medicinal knowledge (TMK) is argued to be a result of experimentation and research, trial and error, providing room for innovative local knowledge learning in local practices and systems, and even incorporating external knowledge based on different worldviews. TMK learning transmission is seen as an informal avenue of learning skills, knowledge, and values between and among generations. In this study, we come across intense learning exchange processes between apprentices and trainers, mostly in the form of oral narratives and practices in and around the local settings and communities. Few studies have looked at the importance of place and space in traditional medicinal knowledge learning processes. There are some recent important contributions by Lindstrom and Muñoz-Franco (2005), who studied the impact of out-migration on certain types of health knowledge, and other researchers who point out how place and social networks are crucial for health knowledge transmission (Andrzejewski et al., 2009).

Within institutional contextual learning arenas, emerging discourses occur, however, studies show that learning processes on TMK in, for instance, South Africa are formalized, which is not the case in the study area in Kenya and Tanzania.

In most parts of Africa, traditional medical practitioners far outnumber practitioners of western medicine within the health sector. However, despite its current importance and common use, traditional medicinal knowledge (TMK) is facing a series of challenges. Societal changes such as rapid urbanization and migration of the younger generations undermine previous TMK learning processes, and certain wild medicinal plants are becoming scarce due to changes in land use and reduction of biodiversity. Odore (2002) further argues that the erosion of people's knowledge associated with natural resources might even be a greater threat than the erosion of the natural resources themselves. Dynamic changes in societal processes in the communities are linked to migration and urbanization.

Intergenerational transfer of knowledge of indigenous herbal medicine is entirely oral among most African communities. In many African societies, traditional and modern health systems exist side by side and people consult both systems though for different reasons and during different stages of the disease (Hountondji, 2002). The education and learning processes of TMK encompass a system of knowledge that involves harvesting, mixing, grinding, drying, knowing symptoms, diagnosing, training and teaching. It includes obtaining proficiency in the identification, preparation, conservation, management, and administration of medicinal products.

An intense learning exchange process occurs between trainee and TH, mostly in the form of oral narrations and practices in and around the homestead. The TMK learning system places a central emphasis on the correspondence of their learning with spiritual invisible entities. Diffusion of health knowledge takes place because of the migration strategies of trainees, and it was quite common for trainees and apprentices to relocate and establish their work in other parts of the country on completion of their training.

The purpose of the present study is to examine how the learning processes of TMK are affected by on-going socio-spatial transformations and migration in Mwanza (Tanzania) and Nyanza (Kenya) in the Lake Victoria Region. The intention has been to investigate socio-spatial aspects of major inter-generational forms of learning of TMK, based on how traditional practitioners themselves define and describe changes in learning processes and the challenges they are facing.

The data and research method applied in order to answer the research questions was a qualitative approach which included semi-structured interviews with both closed- and open-ended questions, focus group discussions as well as participant observations, and literature reviews.

The structure of the study comprises; a methodological and analytical approach to access the data assembled; the theoretical framework in which the research transfusion/diffusion of this knowledge system is embedded: a results section that is developed from the themes which arose out of the empirical data that was collected and analyzed; and a discussion section with conclusions and some recommendations.

The main research questions are:

1. What are some existing perceptions regarding the status of TMK learning and transmission and sustainability in knowledge and passing it on to younger generations?
2. How, where, and when do they teach traditional knowledge to new generations, and what changes are taking place?
3. How do they access non-continuity of learning processes of TMK due to influences of migration that relate to changes in land use and plant availability?
4. How do they see the relationship between TMK and formal knowledge systems and how do they envisage the future of TMK practices?

## Data and Methods

I concede that one form of qualitative methodology could be a way to access perspectives on TMK learning and issues surrounding this learning system. Fieldwork was conducted in Mwanza and Nyanza for a total of 2 months between June and September 2007 and a follow-up fieldwork for 2 months from June to August of 2009. The interviews were conducted during the first period of 2007. The second period is when the FGDs and participant observations took place. The informants were practitioners of traditional medicine, including birth attendants/midwives as well as representatives of TH associations. In addition to interviews and FGDs, participant observations took place during activities of non-timber forest products (NTFP) gathering, healing ceremonies, and market days. Twenty traditional practitioners (THs), 10 men and 10 women, aged between 30 and 95 were interviewed. Interviews lasted between one to several hours, and some THs were interviewed on several occasions. The majority of the traditional practitioners had primary level education, (only two were not educated in the formal education system) while three women and seven men had secondary formal education. In addition to these individual in-depth interviews, five focus group discussions (FGD) were conducted in Mwanza and Nyanza of which one was all female (seven discussants), one all male (seven discussants), and three were mixed (in each of three FGDs, four women and four men). Together with the field assistants, we worked through the initial research questions in order that the questions we posed during the interviews could be suitable for the local contextual sense. It was important that the questions would be translated into the local dialect and then asked by myself/ and or the field assistant. Therefore, substantial time was spent initially prior to the actual interviews and FGDs in developing questions that were directly the research questions.

We were sensitive to the fact that TMK is scientific knowledge that is closely guarded and practitioners often share this knowledge primarily only with individuals who are either close relatives and/or are to inherit the profession. Outsiders are perceived with suspicion. Throughout the fieldwork, we were careful to establish ethical consent with participants, the research board, and other collaborators. The author originates from the region and has older family members who are well-known in the area, which facilitated access and permitted a snow-ball sampling technique for locating THs both in Mwanza and Nyanza. The reliability of the data was accessed through the use of comprehensive data sets from the discussions with National health institutions working with the practitioners for instance the Kenya Medical Research Institute, Institute of Traditional Medicine at the Muhimbili University Teaching Hospital, and NGO organizations working with the THs, e.g., HUPEMEF and Moringa NGO. The traditional practitioners in the study area are organized in associations and organizations which work with local NGOs and in the case of Mwanza with the local district hospital in Magu. The NGOs and local-based organizations provided material that aided me in understanding the linkages between formal and informal health organizations. The data was constantly tested and compared to ascertain the accuracy, while throughout my transcribing of the recorded material, I tabulated and recorded the data in Tables in order to ascertain the accuracy of the data. The field assistants were constantly collaborating with me on my data collection and data analysis assisting in clarifying proverbs, metaphors and other meanings that were unclear to the author. One of the THs whose father had been a prominent practitioner for decades helped in clarifications of proverbs and meanings within the narratives. Interviews were taped and conducted in English, Kiswahili, and Kisukuma in Mwanza, and in English and Dholuo in Nyanza.

During my translation/transcribing exercises (from Dholuo to English, Kiswahili to English, and Kisukuma into Kiswahili then into English) as well as through reading the texts of the transcribed interviews, important themes and subthemes emerged. Braun and Clarke (2006) describe thematic analysis as a method not based on a specific or pre-existing theoretical framework, but rather one used to identify, analyze, and report themes that are closely related to the empirical data. The advantage of the thematic approach is its flexibility and sensitivity to emerging themes in the empirical data. Thematic analysis (TA) was used as a strategy for analysis of interview data, TA is viewed as the main pathway of qualitative methods, due to its path of common approaches with a number of qualitative methods in the social sciences. I thus used thematic analysis to extract themes from my interview data. This approach is sensitive to emerging themes in the empirical data and permits flexibility in terms of theoretical perspectives (Braun and Clarke, 2006). TA describes an analytical approach to the empirical material as a response to the questions and aims of the study. The themes thus identified were closely linked to the empirical/interview material (Braun and Clarke, 2006). The choice to use a thematic narrative analysis approach in the analysis of my empirical material permitted me to reach a more profound understanding of the phenomena embedded in TMK and related practices, as perceived by THs and other respondents. I found it useful to try to find common thematic elements across the narratives and stories represented in the texts transcribed from the interviews and the events they reported. Most of the respondents used proverbs, metaphors, gestures, and “sayings” in their descriptions of phenomena and events in their communities. Major themes related to socio-spatial aspects of intergenerational learning processes were identified and analyzed within a relational understanding of migration in place and space.

## Literature Review

### Research on Inter-generational Learning Transmission

Learning TMK includes obtaining proficiency in the identification, preparation, conservation, management, and administration of medicinal products. While rather few studies have looked at the importance of place and space or socio-spatial dimensions for medicinal learning processes, there are recent important contributions by Lindstrom and Muñoz-Franco (2005), who studied the impact of outmigration on certain types of health knowledge, and other researchers who point out how place and social networks are crucial for health knowledge transmission (Andrzejewski et al., 2009). Through several generations, knowledge on the identification of plant species with medicinal properties and their use has been developed (Sheldon and Balick, 1995). TMK learning is viewed as both temporal and spatial or place-based. It relates to language, historical processes, and social relations which are largely influenced by political, economic, and social processes (Hanks, 2007). In line with a relational understanding of social and spatial dimensions, intergenerational learning processes of medicinal knowledge are in this study understood as place-based and related to history, language, and social relations (Geissler et al., 2002). Some studies discuss prolepsis which takes a socio-cultural theoretical approach that conceptualizes the transmission of knowledge between generations where experiences are passed down and knowledge and values re-evaluated in the context of a rapidly changing world (Cole, 1996). Studies on learning processes that are inter and intra-generational discuss processes that are co-constructed within relationships of mutuality and reciprocity (Eyssartier et al., 2008), while the transmission is generally oral and contextual (Mashelkar, 2002). Inter-generational relations and the priority accorded to seniority, which is at the core of social organization in Africa, have determined the modalities of learning processes of younger generations. Given the numeric importance and the heterogeneity of the young demographic group, these processes change relative to the social context (French Institute for Research in Africa, 2006). Prince and Geissler (2001) describe how traditional medicinal knowledge among the Luo is usually imparted between alternate generations. These skills are also seen to complement formal educational learning skills while invoking cultural continuity and change (Kenner et al., 2007).

As is the case for the Luo in Kenya (Sankan, 1995; Sindiga, 1995) and the Wasukuma of Tanzania, oral transfer of knowledge of ethnomedicine is also common in other ethnic groups in East Africa (Ochieng' Obado et al., 1995; Sindiga, 1995). Training to become a TH usually starts during the pre-adolescent age when the child is perceived as receptive, obedient, has a good memory, and can keep secrets (Mwiturubani, 2009). Luo plant medicine has been argued to be mainly a domain of women's activity (Olenja, 1991) but in general, in the study region, both men and women engage in TMK learning processes, while the three major forms of becoming a TH—inheritance, own illness, and calling—are not gender-specific.

While Battiste and Henderson (2000) argue that knowledge transfer is not perceived in the context of something that some possess and others do not, with a lifelong responsibility and that cannot be controlled by educational institutions, other scholarships view “knowledge as a universal heritage and a universal resource while emphasizing its diversity, variation and being context-specific” (Hountondji, 2002). Mejeke argues however that the present system of education in Africa emphasizes social and cultural contexts that are far removed from conceptual structures that are within African communities (Majeke, 2002). A fundamental transformation with an aim of altering educational syllabi can be seen in what is described as mutual decolonization (Crossman and Devisch, 2002).

South Africa has developed an institutional model of TMK of the Sangomas (THs). Education within schools provides students with learning arenas where they graduate and are able to practice their profession as sangomas (Thornton, 2009). Increased mobility and rural-urban migration by individuals to townships and cities in search of livelihoods and opportunities are similarly predominantly the case as socio-spatial transformations continue to evolve in the study area.

Similarities exist between the Luo and the Wasukuma in terms of historical migrations and TMK practices; the early Luo settlers in Kenya also had a pastoralist orientation. The fact that the Wasukuma have proved successful in their migratory movements has been attributed to their use of “powerful” TMK which is considered superior to those of neighboring ethnic groups (Sanders, 2001). Male out-migrations from rural to urban areas have also been characteristic of this region and remittances from migratory wage labor provide important cash income for families left behind. Rural-urban migrations involve social, economic, and cultural transformations, including changes in health practices and knowledge which will influence how TMK is perceived by the younger generation.

### Institutional Settings for Traditional Medicinal Knowledge

Recent ethnographic studies from western Kenya describe Luo medical practice as a pluralistic “landscape of therapy,” in which people move between multiple therapy sources (Geissler et al., 2002). Historically, traditional knowledge systems have been marginalized in relation to western systems (Hoopers, 2002; Hountondji, 2002; Majeke, 2002). Although Colonial governments appreciated the existence of TMK alongside the introduced Western medicine, there was not much effort to promote this knowledge field. Consequent efforts and official policy on TMK after independence have varied and there are important differences in formal and informal perceptions, practice, and policy on TMK between Kenya and Tanzania. In Kenya, this sector is within the national culture and social services sector while in Tanzania it is within the health sector.

The Kenyan and Tanzanian governments' policy for free primary education has provided incentives and opportunities for school attendance in both study areas, which also has led some TPs to promote the combination of TMK with formal primary education. Learning processes occur that combined formal medical education in the urban area and then return to the rural area for training as a TP, thus young people are encouraged to become practitioners by way of combining the profession with formal medical studies.

Majeke (2002) puts forward that contents of syllabi emphasize the social and cultural rhythms of the early (colonial) settler communities with conceptual structures and categories of thought borrowed from European days of the past. Colonial authorities taught and trained indigenous African students in schools and tertiary institutions in skills that did not fit them back into their communities, and that forced them to work in employment situations where foreign people's undertakings were situated. For trained medical doctors and most health professionals, it often meant working in “modern” sections of the urban areas.

Unlike the case of the institution of traditional healing of the *sangoma* in South Africa, where knowledge transmission takes place within schools from where students obtain membership and graduate as *sangomas* (Thornton, 2009), the learning processes by traditional practitioners in Kenya and Tanzania are not organized in a formal system of education in specific locations. Traditional practitioners nevertheless have their professional networks, organizations, and meetings, for instance within the Traditional Practitioners Association in Homabay and Traditional practitioners Association in Mwanza. Official documents in Kenya and Tanzania state that ongoing socio-spatial dynamics could be transforming the role of the THs in the study region (see e.g., the National Health Policy in Tanzania, the National Strategy for Growth and Poverty Reduction of Tanzania 2011, and the Draft National Policy for Traditional Medicine and Medicinal Plants in Kenya 2008), but they do not tell the story at the grassroots level, and less still the perceptions of the THs.

## Results

### TMK Learning Processes and Socio-Spatial Changes

The empirical material showed a range of learning practices of practitioners, who worked mostly in their own houses and had their teaching organized spatially according to the location of plants and places of special significance. The practitioners described the practical ways by which TMK is transferred gradually, over a long period of time and developed from the knowledge of one kind to different varieties and types of medicinal products. The main approach of training is learning by doing in the home of the TH. This usually requires that the trainee lives in-house and has time to “sit” with the TH, most often in the rural area, sometimes at a far distance from primary schools, and during prolonged periods of time. The trainee repeats the different healing procedures until he or she is an expert, and it may for instance take years to remember the names of the numerous (sometimes over 200) different types of medicine:

You see… in training for traditional native medicines, they say how come you have managed all diseases? Because you have sat on it for years… see this young boy, he has been here since he was a small boy and he is still in training (male TH Magu).

While oral narrations are central in the training, THs place little importance on written information. One TH described how written information may even be less likely to be viable as enabling the transfer of knowledge than oral training:

The 1,000–2,000 trees I have planted… I tell them to go and pick the trees and when they come I show them which and which to mix together. That is how we teach those trainees. If you keep it in the book, nothing! It will get lost (male TH Magu).

This shows the importance given to “sitting” with the TH in order to learn the plants, rather than “keeping them” in books. Resident trainees were more common in Magu, Tanzania than in Homabay Kenya and more often in the rural than urban settings. Trainees who reside with the THs within the urban setting are most often (though not always) the direct offspring of the TH. When the trainee attends formal education, he or she goes to the TH's practice after school and undertakes further training during the school holidays. THs expressed concern about rural-urban migration and changing livelihoods of the younger generation, which apart from less time for learning could lead to negative values and attitudes toward TMK:

My son was taught by his grandmother's sister but he has left this work and does not attach value to it (male TH Suba, Kenya).

The young people are not vigilant and are not interested. They think it is old-fashioned and only prefer modern medicine (female TH Rachuonyo, Kenya).

However, the interviews also showed that young people increasingly realized the income potential of traditional medicine through observations of the marketing of the products in the urban areas. Traditional practitioners increasingly sell their products and provide their services in urban markets:

The youth are interested when they see that I have an income; I sell at the market in Homabay and at Rodi Kopany (male TH Gem, Kenya).

This male traditional practitioner observed that when young people saw that he could “make a living” from marketing the products, they showed a keen interest in studying traditional medicine. This interest however faced challenges as the learning processes themselves require a spatial setting where the trainee can “sit” with the TH for prolonged periods of time and have access to recollection areas of medicinal plants. The interviews showed that tougher socio-economic conditions both in rural and urban areas make it more difficult for TPs to provide housing for trainees, and, obviously, school attendance makes time more limited for a learning practice that takes many years even when it is continuous. The importance of teaching indigenous knowledge to the youth was highlighted by the practitioners in both rural and urban settings, but they also stressed the challenges to TMK related to migration and rapid urbanization.

### Being Sent

“Being sent” is a widespread practical way of learning the characteristics and availability of plants and a form of learning by doing which is at the core of TMK. The trainee is sent to specific destinations to collect the products. Being sent is emphasized as crucial for obtaining the knowledge, but is also a form of payment from the trainee to the TH. The trainee is regularly sent to the forest or bush to collect and harvest medicinal plants in order to bring them back to the homestead, which can be both time-consuming and tiring.

First, if you want to know about work you should be a person's messenger. She says, go and dig this medicine, you see this medicine… go and look for it and bring it to me… so you do it until you will know it (female TH Gem).

The TP shows the trainees the exact character of the medicines explaining what they cure. Being sent and in-house repeated demonstration and practical work with patients are the ways the trainee receives the education. Without fees paid for the education, the trainees contribute as a form of payment to be sent to harvest the medicine, help on the farm, and provide other services within the homestead.

Is there any payment they give you? No, they do not pay me anything… so what benefits will you get from showing them? My interest is that I give them. They acquire the legacy from me. I want them to acquire the knowledge from me (male TH HB).

According to the stories of the practitioners, the teaching, transfering, and processing of TMK today have both similarities and differences with the ways the older generation learned their practices. Some major differences relate to the abundance of plants closer to the homestead in previous times. The geographical distances to places of harvest as well as to beneficiaries have increased, which means both that trainees have to be sent long distances and that new plant preservations techniques have developed:

It is not different, but the style in which I use the medicine… is different from the old time, the system is different. You know, the old people used to dig the medicine, put it in a pot and boil it, and then people drank it. I take the medicine, I pound it until it is very soft, soft… then I spread it in the sun and it dries and I use it in powder form. It means that in powder form it can be used for a long time, you know, it can last longer while the boiled one has a short shelf life… (female TH HB).

The youth today do not agree to be sent (male TH Gem).

The older TP's thus harvested, boiled the TM, and consumed it as a liquid or the boiled leaves. Today, the practitioners use drying and pounding techniques, which provide a longer shelf life and ease with transportation. Increased migration to the urban centers, which are situated away from the locations where the medicinal products are harvested, has necessitated a change of preservation techniques to accommodate the longer shelf life of the products. The steady reduction in the availability of medicinal products together with the difficulty of sending trainees long distances also necessitates preservation. Practitioners in both Mwanza and Nyanza described how the older generation used to cultivate TM close to the homestead to help in accessibility and teaching trainees and family members, combining gathering TM from the wild bush with planting them closer to the homestead. Some of the younger TPs do cultivate plants close to the homestead, but informants also described how they had to seek permission and pay in order to be able to harvest from other clan lands. Among the Luo, a man's plots are divided among his wives who if deceased pass them on to male children with the senior son receiving the largest portion (Ochieng, 1997). With the socio-economic and socio-spatial changes, land allocation and accessibility have changed and land has become scarcer. When clan land gets overcrowded there is further migration to found new polities elsewhere (Ochieng, 1997). This indirectly or directly influences harvesting and cultivation practices of traditional medicine and the empirical data revealed that this was particularly the case in the Nyanza context where scarcity of land is more apparent than in Mwanza.

### Ritual Places

Prayer and rituals form an important part of the trainees' education. One “prays to the ancestors for guidance to find the medicine” as one TP expressed it. Larger rituals are an integral part of a healer's work and they are carried out periodically (bi-annually; every 3 years). The knowledge of rituals is taught during the learning period and the specific ritual differs from person to person. Some ritual ceremonies use staple fodder and animal products (milk, ghee, sorghum, and millet) adorning a special dress code for all participants. Almost always, a special tree has been chosen as the venue for the ritual ceremonies and these trees are usually situated hundreds of km away from the THs home place. These trees are often not available locally due to deforestation; the specific tree species are rare and often situated at long distances. These sacred places are visited to acquire spiritual power, perform rituals, and collect medicines. One example from the interviews was one healer who traveled with the trainees ≈700 km from Magu to the Tanga Region in Tanzania to perform the ritual. As the tree is situated in another region there must be an agreement with the local village council to enable the visitors to carry out their work. The rituals and ceremonies in specific sacred places were a more important function in Mwanza than in Nyanza. Due to increased migration dynamics in both contexts of younger populations moving to the urban places in search of alternative livelihoods, this form of training is becoming increasingly rare for the youth given the long distances to the ritual sites, which involves many days' travel.

### Migration and Health Knowledge Diffusion

In contrast with TPs who inherited the gift at a young age, there were those who learned TMK through their own illness and/or started training later in life. Through the cure of a prolonged ailment, some chose to become practitioners themselves after a period of training with an older TH for up to 3 years. The suffering itself was then seen as part of and even a requisite for the learning process.

I was hurting. After I was healed I started to treat others one by one (female TH, Magu).

The research revealed that becoming a TP at a later age, sometimes through own illness, frequently took place outside the home area of the trainee, and often even outside the country of origin. After graduation, a number of these trainees migrate back to their original homes and set up their own village hospitals. The latter was seen more often in Tanzania than in Kenya.

We have given many who now have their own villages, more than 10 persons, they are now in Dar es Salaam, Musoma, Tarime… (…) and also Kenya (male TH Magu).

Those who I am giving the system, they may in the future provide even better ways of treating and having a central role, perhaps they will be able to treat even better than I do, make TMK even have a bigger role, they may improvise (Male TH 67 years. Homabay, Kenya).

Trainees migrated temporarily to undergo treatment and/or learn TMK with the aim of returning to their home region to practice their trade there. In conversations with older TPs, they expressed how the traditional medicinal knowledge is not “owned” by them, but given to them by different venues as a gift from a higher deity, which they in turn are to give to others. The gift is supposed to be used to help cure ailments and societal problems. It is the duty of the traditional practitioner to act as a medium through which this gift is shared with individuals within the society who may need it, thus diffusion of the knowledge is central. However, TPs should only teach TMK to trainees whom they perceive to have a “good nature,” who can “do good,” and have empathy for their fellow human beings, which also will lead to new discoveries in the medicine:

You see someone whose heart is good (…) You do not just give it to anyone… if you see someone who is hurting then you have sympathy for helping him… so then a lot of discoveries can come out of that medicine for you … (female TBA Homabay).

Those who I am giving the system, they may in the future provide even better ways of treating, they perhaps will be able to treat even better than I do, there may be improvising (male TH HB).

Negative suspicions were expressed by some traditional practitioners who perceived that if they trained the wrong person he/she might be dishonest and take the knowledge and sell it for private benefit. The person may move and “take their knowledge and their customers” and use it for their own interests, indicating that “some could even use it to harm others.” According to some respondents, there are a number of practitioners who may want to replicate what they do, claiming knowledge of the medicines they have. TPs linked this negative knowledge diffusion to outmigration, with potentially negative effects on patients:

Adding someone's knowledge as it was added to me, I still find it difficult in one way. There was someone with a good idea and they took him and gave him a job. Then it happened that he was sent away from the work. Then you know that those people have remained with all his ideas…and then they take the customers that you used to get (male TH Gem).

Many herbalists think the medicines which I have, they should also have, so they take them…at times they give wrong medicines and overdose them, which can injure people (male TH HB).

The discussion on sorcery arises in the empirical data, particularly within the context of ethics and socio-cultural and socio-economic problems. In all interviews, this phenomenon was mentioned and vehemently criticized by the TPs and authorities. In the citations above, the fears were in particular related to the mobile younger generation's uses of TMK. Dynamic changes in societal processes in the communities linked to migration and urbanization highlighted the role of parents, who sometimes feared and critiqued the TP's work:

Parents think it is negative and they have fear (male TH Gem).

Some fear and accuse the young people of learning how to bewitch and kill people… (male TH Gem).

The family and household need to have a consensus on if the TMK can be taught to the youth (female TH Gem).

Traditional practitioners thus expressed concerns in relation to knowledge diffusion through increased migration, but there were also those who saw both livelihood opportunities and possibilities of closer interactions between TMK and “modern” medicine in light of increased urbanization. Some of the TPs who were interviewed had future plans to expand premises for patients both in rural and urban areas. In particular in Tanzania, commonly mentioned were plans to cultivate medicinal plants on land already purchased and acquired for this purpose. Some TPs saw the way forward in finding new ways of combining TMK learning with formal education, thus bridging rural practices with urban educational and market opportunities.

### Rural–Urban Migration Tensions and Interactions of TMK With Formal Knowledge

While expressing concerns about the future generations, many respondents nevertheless stressed that youth are interested and wish to practice as TPs. We found learning processes that combined formal medical education with TMK, and some traditional practitioners, who themselves had formal western education, encouraged their offspring to complete their education before pursuing work with traditional medicine.

Are the youth interested in learning about traditional medicine? Very much. The moment they learn this they want to continue…I say they should finish school first, and to those who have finished, I teach the treatment (male TH HB).

There is another one who tried to read, and recently went to the college of medicine. Now he has finished and is at home… you see he has inherited (male TH HB).

I used to teach both modern medicine and traditional medicine. People come and I also refer to the hospital. Every 2 days they come and I give advice (male TH, Suba).

In our study area, we found several cases of medical pluralism (We understand medical pluralism as the consultation of both traditional and western medical practices). Some Wasukuma practitioners explicitly recognized the benefits of modern medicine, and several of our respondents suggested that there are certain ailments that only a traditional healer can cure, yet there are other sicknesses that a modern hospital can more readily heal with technologies such as intravenous fluids and store-bought medicines. Pragmatic considerations were common, but forms of true cooperation between the two systems were rare. The interactions between traditional and modern health systems were related to rural–urban inequalities and did not take place without complications. We found only a few cases of close cooperation between practitioners and modern health systems, such as when the TP referred his patients to hospitals, and one practitioner received patients from the hospitals and organized a transportation system to facilitate the interaction between his village and the urban hospital. In contrast with this positive interaction, facilitated by practitioners' knowledge of both traditional and “modern” medicine, there were often pessimistic views among traditional practitioners about the future of TMK and the role of the younger generation in endorsing it. The growing disenchantment with farming as a way of life has made young rural based people in both Mwanza and Nyanza to migrate and actively diversify into non-agricultural activities. Rural-based TMK is not perceived as a viable long term livelihood strategy for the younger generation, but some traditional practitioners envisioned a strategy for young people to become practitioners by way of combining formal medical studies in the urban area and then return to the rural area for training as a TP. Two respondents had sons who were attending urban formal education and intended to complete it before continuing working with traditional medicine in the rural setting. Another respondent's son, who was 18 years old, had decided to become a doctor in formal medicine and thereafter practice as a traditional healer. TPs testified that it has become more common that younger TPs are trained in both systems.

## Discussion

The younger populations attribute lower value to TMK which indicates rising challenges of TMK and its transmission to further generations of TPs. With the introduction of formal western education during the colonial and postcolonial eras, there was a disdain for traditional knowledge, and children were expected to abandon previous learning systems (Miller, 1996). Despite the continued use and importance of TMK, this legacy contributes to prevailing negative perceptions and suspicions about learning TMK. The traditional practitioners interviewed in this study described how environmental pressure, migration of the youth, and socio-spatial changes in the study area over the last three decades have created new challenges for TMK practices. Some were concerned about negative values about TMK in the younger generation, while others stressed the will of young people to engage in training and become practitioners. The youth's keen interest to learn was seen to increase when they viewed improved livelihood possibilities of THs due to the commercialization of medicinal plants, especially in the outmigration spaces.

Some of the interviewed practitioners pointed out the missing link between TMK learning processes and the formal education system. Baimba (1993) argues that “traditional knowledge learning which makes sense of the world to traditional students, often conflict with what is taught in science lessons. Hence for the majority of these students, the “meaningless” information provided in the science lessons is shelved, to be used only for the purpose of passing school examinations. Once outside the school, it is the traditional knowledge which they use to make sense of their world” (Baimba, 1993, p. 28). Our study showed a strong influence of modern education in affecting the perceptions and access of the youth to TMK. With this opportunity, the youth who migrate to attend modern education have limited time if any to learn TMK. The future of TMK learning processes may be limited unless incentives are in place for the youth, regarding their future livelihoods. Odore (2002) argues that in Africa, colonial science and education are knowledge *on* Africa. The problem today is how to make it knowledge *by* Africans for their own collective promotion and development. This is a context in which traditional medicinal knowledge is “barely tolerated and exists in subjugate deference to a mainstream form of knowledge that is promoted as the only way of seeing, and the only tool by which people can receive accreditation and a license to *be”* (Odore, 2002, p. 14). The Wasukuma and Luo's youth livelihoods are increasingly merging into circumstances that place a lower value on their traditional medicinal knowledge. Under this pressure, traditional knowledge of medicinal plants is starting to disappear, with little to take its place. Formal knowledge is commonly promoted to young people but too often without providing the means to gain access to it (Beyer, 2009).

The study showed that the role of TMK in the past was very central to community health care and that it continues to be significant. The interaction between traditional practitioners and the modern health system varied in the different places of the study area, with examples of close and uncomplicated cooperation in some places and little or no interaction in others. In both study areas, the THs generally stated that there are some signs of a new awareness and popularity of TMK, but the younger generation does not take TMK as seriously as the older generation and there is a need for concerted efforts for its promotion and youth involvement.

A central question during the interviews with practitioners was how young people will be taught in the future. During fieldwork, it was not uncommon that there were no trainees in the homesteads of THs. Many young people lack interest in learning TMK and do not approach them often, but in both study areas, there were TPs who had trainees who were positive and interested in learning. If assistance were provided, a number of TPs mentioned that they would in the future be enabled to organize more training for the interested youth. The youth who are receiving this TMK would be better equipped if combining TMK with modern medical knowledge and, as one interview person expressed it, might be able to improvise some of the ways in which they treat.

The prevailing dominant scientific paradigm in school education is a context where few elements of TMK practices are permitted to surface (Indigenous Knowledge and Peoples Knowledge (IKAP), 2007). The youth, who migrate between these two knowledge systems, take action out of the predominant worldview, as seen in the study. Tensions between the youth and elders emerge, knowledge is lost and undermined, while biodiversity is threatened and diminished. Some researchers argue that the increased migration of youth to urban centers denies the younger generation traditional community support systems, which include education in survival skills, communication skills, safety, and conflict prevention (Ntuli, 2002).

Also noted is that as well as being ancient, TMK is modern for these communities as it reacts to changes and is open to learning (for instance, in relation to HIV/AIDS mitigation). TMK is a result of experimentation and research, trial and error, providing room for innovative local knowledge learning in local practices and systems, even incorporating external knowledge based on different worldviews. Some TPs are not enthusiastic to share information regarding their TMK, partly due to suspicion that the knowledge will be “pirated” for private profit but also due to false TPs who may claim knowledge of the medicines while giving customers wrong doses which can prove fatal to the users.

In both urban settings of the study area, TPs have established associations of TMK practitioners in which many of our interviewed persons were members. The major challenges revolve around their roles and relationships with the formal medical establishment as well as issues related to socio-spatial changes such as increased rural-urban migrations, and biodiversity loss. Colonial structures are perceived to have been detrimental to the social dynamics of TMK as these structures negated traditional knowledge and subordinated it. Despite this legacy, most TPs could see new roles of TPs and emphasized the promotion of TMK as a continued important aspect of community health in response to rapid socio-spatial changes and outmigration dynamics.

## Data Availability Statement

The raw data supporting the conclusions of this article will be made available by the authors, without undue reservation.

## Ethics Statement

The studies involving human participants were reviewed and approved by Tanzania National Commission of Science and Technology. Written informed consent for participation was not required for this study in accordance with the national legislation and the institutional requirements.

## Author Contributions

The author confirms being the sole contributor of this work and has approved it for publication.

## Conflict of Interest

The author declares that the research was conducted in the absence of any commercial or financial relationships that could be construed as a potential conflict of interest.

## Publisher's Note

All claims expressed in this article are solely those of the authors and do not necessarily represent those of their affiliated organizations, or those of the publisher, the editors and the reviewers. Any product that may be evaluated in this article, or claim that may be made by its manufacturer, is not guaranteed or endorsed by the publisher.

## References

[B1] Andrzejewski C. S. Reed H. E. White M. J. (2009). Does where you live influence what you know? Community effects on health knowledge in Ghana. Health Place 15, 228–238. 10.1016/j.healthplace.2008.05.002 18603464 PMC2614676

[B2] Baimba A. (1993). The scientific knowledge/cultural interface: implications for science education in non-western cultures. J. Sci. Teach. Assoc. Niger. 28, 13–19.

[B3] Battiste M. Henderson J. Y. (2000). Protecting Indigenous Knowledge and Heritage: A Global Challenge. Saskatoon, SK: Purich Publications.

[B4] Beyer S. (2009). Singing to the Plants: A Guide to Mestizo Shamanism in the Upper Amazon. Albuquerque, NM: University of New Mexico Press.

[B5] Braun V. Clarke V. (2006). Using thematic analysis in psychology. Qual. Res. Psychol. 3, 77–101. 10.1191/1478088706qp063oa

[B6] Cole M. (1996). Cultural Psychology: A Once and Future Discipline. Cambridge, MA: Harvard University Press.

[B7] Crossman P. Devisch R. (2002). “Endogenous knowledge in anthropological perspective – a plea for a conceptual shift,” in Indigenous Knowledge and the Integration of Knowledge Systems Towards a Philosophy of Articulation, ed C. A. Odora Hoppers (Claremont: New African Books), 96–127.

[B8] De Bruijn M. Van Dijk R. Foeken D. (2001). Mobile Africa, Changing Patterns of Movement in Africa and Beyond. Leiden: BRILL. 10.1163/9789004492202

[B9] Eyssartier C. Ladio A. H. Lozada M. (2008). Cultural transmission of traditional knowledge in two populations of North Western Patagonia. J. Ethnol. Ethno Med. 10, 4–25. 10.1186/1746-4269-4-25 19077315 PMC2614966

[B10] French Institute for Research in Africa (IFRA) (2006). “Youth in Eastern Africa: past and present perspectives,” in Conference Paper on Youth in East Africa (Nyeri: British Institute in Eastern Africa), 7–14.

[B11] Geissler P. W. Harris S. A. Prince R. J. Olsen A. Odhiambo A. R. Oketch-Rabah H.. (2002). Medicinal plants used by Luo mothers and children in Bondo district Kenya. J. Ethno Pharmacol. 83, 39–54. 10.1016/S0378-8741(02)00191-5 12413706

[B12] Hanks R. (2007). “Inter-generational learning and the contributions of older people,” in Ageing Horizons, eds Newman S. Hatton-Yeo A. (Oxford: Oxford Institute of Ageing), 31–39.

[B13] Helgesson L. (2006). Getting ready for life: life strategies of town youth in Mozambique and Tanzania (Doctoral dissertation). CERUM Social and Economic Geography, Umeå University, Umeå.

[B14] Hoff A. (2007). Inter-generational Learning as an Adaptation Strategy in Aging Knowledge Societies. Warsaw: European Commission Education, Employment.

[B15] Hoopers C. O. (2002). Indigenous Knowledge and The Integration of Knowledge Systems: Towards a Philosophy of Articulation. Claremont, CA: New African Books.

[B16] Hountondji P. (2002). “Knowledge appropriation in a post–colonial context,” in Indigenous Knowledge and the Integration of Knowledge Systems, Towards a Philosophy of Articulation, ed H. C. Odore (Claremont: New African Books), 23–39.

[B17] Indigenous Knowledge and Peoples Knowledge (IKAP). (2007). Intergenerational Transfer of Indigenous Knowledge and Skills. Chiang Mai: IKAP. p. 5–9.

[B18] Kenner C. Ruby M. Jessel J. Gregory E. Arju T. (2007). Inter-generational learning between children and grandparents in east London. J. Early Child. Res. 5, 219–243. 10.1177/1476718X07080471

[B19] Lindstrom D. P. Muñoz-Franco E. (2005). Migration and the diffusion of modern contraceptive knowledge and use in rural Guatemala. Stud. Fam. Plann. 36, 277–288. 10.1111/j.1728-4465.2005.00070.x 16395945

[B20] Majeke A. M. S. (2002). “Towards a culture-based foundation for indigenous knowledge systems in the field of custom and law,” in Indigenous Knowledge and the Integration of Knowledge Systems Towards a Philosophy of Articulation, ed H. C. Odore (Claremont: New African Books), 141–150.

[B21] Mashelkar R. A. (2002). “The role of intellectual property in building capacity for innovation for development,” in Indigenous Knowledge and the Integration of Knowledge Systems Towards a Philosophy of Articulation, ed C. Odora Hoppers (Claremont: New African Books), 188–199.

[B22] Miller J. R. (1996). Shingwauk's Vision: A History of Native Residential Schools. Toronto, ON: University of Toronto Press.

[B23] Mwiturubani D. A. (2009). Sustaining Food Security in the Lake Victoria Basin through Indigenous Coping Mechanisms to Water Resources Variations. Nairobi: Institute of Security Studies, KM Africa Knowledge, 1–2.

[B24] Ntuli P. (2002). “Indigenous Knowledge Systems and the African Renaissance-Laying a foundation for the creation of counter-hegemonic discourses,” in Indigenous Knowledge and the Integration of Knowledge Systems Towards a Philosophy of Articulation, ed H. C. Odore (Claremont: New African Books), 53–67.

[B25] Ochieng' Obado E. A. Odera J. A. Sindiga I. (1995). Management of Medicinal Plant Resources in Nyanza, Traditional Medicine in Africa. Nairobi: East African Educational Publishers Ltd.

[B26] Ochieng W. R. (1997). Outline History of Nyanza up to 1914. Nairobi: East African Literature Bureau.

[B27] Odore H. C. (2002). Indigenous Knowledge and the Integration of Knowledge Systems – Towards a Philosophy of Articulation. Claremont: New African Books.

[B28] Olenja J. M. (1991). “Women and health care in Siaya District,” in Women and Development in Kenya, Siaya District, eds Were G. S. Suda C. A. Olenja J. M. (Nairobi: Institute of African Studies), 44–70.

[B29] Prince R. Geissler P. W. (2001). Becoming One Who Treats: A Case Study of a Luo Healer and Her Grandson in Western Kenya. Copenhagen: Copenhagen University. 10.1525/aeq.2001.32.4.447

[B30] Sanders T. (2001). “Territorial and magical migrations in Tanzania,” in Mobile Africa, Changing Patterns of Movement in Africa and Beyond, eds De Bruijn M. Van Dijk R. Foeken D. (Leiden: BRILL), 27–46. 10.1163/9789004492202_007

[B31] Sankan S. S. (1995). The Maasai. Nairobi: Kenya Literature Bureau.

[B32] Sheldon J. W. Balick M. J. (1995). Ethno-botany and the Search for Balance Between Use and Conservation. Intellectual Property Rights and Biodiversity Conservation. London: Cambridge University Press. 10.1017/CBO9780511623417.004

[B33] Sindiga I. (1995). Managing Illness Among the Luo. Traditional Medicine in Africa. Nairobi: East African Educational Publishers Ltd.

[B34] Thornton R. (2009). The transmission of knowledge in South African traditional healing. J. Int. Afr. Inst. 79, 17–34. 10.3366/E0001972008000582

[B35] World Health Organization (WHO) (2002). Traditional Medicine Strategy, 2002–2005. Geneva: World Health Organization.

